# Operative Gynecology

**Published:** 1899-09

**Authors:** 


**Operative Gynecology.** By HOWARD A. KELLY, M.D. Vol. I., pp. xvii., 563. Vol. II., pp. xiii., 557. London: Henry Kimpton. 1898.

We have frequently in these columns expressed our admiration of the work of those associated with the Johns Hopkins Hospital, and Dr. Kelly's admirable book reflects the highest credit upon himself and the institution with which he is connected. The object of the writer has been to set forward his own experiences, and to record those methods of procedure which he has found to be trustworthy and best, and we hail with the greatest satisfaction the ability to read for ourselves the masterly exposition of one so famed in the gynæcological world as the author. The work is large, but a considerable amount of space is occupied by the twenty-four plates and five hundred and fifty original illustrations with which it is embellished. Of the letterpress as a whole we may say at once that it is clear and incisive, and free from verbiage, carrying its own conviction as the work of one whose experience and judgment are of the highest order. The illustrations too are masterpieces of execution, and render the book one of the most beautifully adorned works of its kind.

In the first volume, the three opening chapters deal mainly with the question of sepsis, antisepsis, and bacteriology. Every detail of the disinfection of the patient, surgeon, and appliances is carefully described, and a brief account is given of the chief organisms (and the methods of staining them) met with in gynæcological practice. For disinfection of the hands before operation the author prefers the use of permanganate of potash and oxalic acid. Then follow chapters on topographical anatomy, the method of making a gynæcological examination, the use of local and general anæsthesia, and the general principles involved in plastic operations.

Chapter IX. treats of diseases of the external genitals, and several plastic operations for the removal of large portions of these parts for tubercular disease, pruritus vulvæ, &c., are described. The plastic art of the surgeon is still further brought out in Chapter X., which deals with rupture of the recto-vaginal septum and relaxed vaginal outlet, and here we can find a lucid account of the true principles underlying a successful operation for these conditions

—an operation which has suffered much from the incorrect and unintelligible descriptions which are often met with. Operations on the vagina is the subject of Chapter XI., the most important being those necessary for traumatic affections as the result of injuries incurred in labour. Chapter XII. treats of affections of the urethra and bladder, and Chapter XIII. of affections of the ureters, and it is in the diagnosis of these conditions in the female that Dr. Kelly has worked so laboriously and successfully in perfecting his methods, which are universally recognised to be of the greatest value. These methods, which have long been known from his other writings, have been largely instrumental in making a reputation which is world-wide and well-deserved. In connection with large vesico-vaginal fistulæ, we are glad to see that the author regards the operation of kolpokleisis as a thing of the past. The final chapters (XIV.—XIX.) deal with operations upon the cervix of the uterus, including dilatation and curettage; prolapse of the uterus; vaginal hysterectomy; vaginal extirpation of submucous myomata and polypi; and certain conditions in which the uterus acts as a retention-cyst. The account of the examination of uterine scrapings after curettage bears witness to the care and exactness with which Dr. Kelly's work is performed. We regret that nothing is said of the results of vaginal hysterectomy for cancer, for the author's large experience and opinion would have been of great value in estimating the much-discussed value of this operation.

The second volume opens (Chapters XX.—XXII.) with an account of the general principles and complications common to abdominal operations; the care of wound and patient up to recovery; and complications arising after abdominal operations. These chapters deal in a most complete manner with the subjects of which they treat, and give much reason for reflection, and, if space permitted, for discussion also. Each surgeon doubtless falls into certain routine treatment in the after-management of abdominal sections, and consequently the practice of others may appear to be wanting in certain ways. Hence we feel disposed to point out the great value which we attach to the treatment of tympanites by turpentine enemata, and to the use of strapping over the dressings to support the abdominal muscles as it were in a splint, neither of which appears to find a place in the author's practice. Of still greater importance, however, is it for us to raise a protest against the restriction of all fluids by the mouth for the first twelve hours or more after operation. This would seem, in face of the intense thirst which often exists, to amount to an unnecessary piece of torture (the word is not too strong in many instances), for we have long been in the habit of allowing tablespoonful doses of hot water at frequent intervals, and can unhesitatingly say that we have never known it productive of harm. Chapter XXIII. deals with tubercular peritonitis, and we note with satisfaction that the author agrees in abandoning all drainage in these cases. In Chapter XXIV. the author describes his method of suspension of the uterus by attaching the posterior surface of the uterus to the abdominal wall, and though we cannot accept this as the best operation, we would admit that the author's experience has shown that it is less open to objection than many others. Chapter XXV. treats of conservative operations on the tubes and ovaries. This is one of the signs of the times, and is an important contribution to the subject which all operating surgeons would do well to consider. Salpingo-oöphorectomy is considered in Chapter XXVI.; vaginal drainage and enucleation for pyo-salpinx, &c., in Chapter XXVII., and abdominal hystero-salpingo-oöphorectomy in Chapter XXVIII. To those who are not familiar with the life-saving advantages of vaginal drainage in certain cases, this portion of the book may be commended. Ovariotomy is the subject of Chapter XXIX., and abdominal hysterectomy for carcinoma of the uterus is the subject of Chapter XXX. In the latter chapter the author gives excellent reasons for his preference for the abdominal rather than the vaginal route, and after giving certain rules for the prevention of cancer, he says: "If these rules were conscientiously observed, there can be no doubt but that thousands of lives would be saved yearly in this country alone,"—a statement which amounts to a grave (and not altogether undeserved) indictment against both the profession and the public which it is well that both should consider. Chapter XXXI. gives an account of myomectomy, and hystero-myomectomy, and the latter operation is confined to the retro-peritoneal method. Chapters XXXII.—XXXIV. are taken up with operations during pregnancy, Cæsarean section, and extra-uterine pregnancy, and Chapters XXXV. and XXXVI. deal with the radical cure of hernia and intestinal complications. These last cover a wide area, and are comparatively briefly treated, and rightly so, for they only indirectly concern the gynæcologist. The final chapters (XXXVII. and XXXVIII.) describe the more remote results of abdominal operations and the conduct of autopsies.

We have spoken of the contents of the book in some detail, to show what an extensive ground is included thereby. Such a work must long remain as a standard book of reference, and will always be a lasting memorial of the labours of the author both in deed and word. The one weak spot is in the index, which might be considerably enlarged, and so further enhance the value of a work of which we have little to say except in appreciation.

